# On the Etiology of Certain Cases of Alopecia

**Published:** 1919-11-15

**Authors:** 


### THE NEW DERMATOLOGY.

### IV. On the Etiology of Certain Cases of Alopecia.

IN this article it is not proposed to deal with the common form of baldness ("calvitie" of the French), which is seen chiefly in the male sex, but with the alopecia following acute illness, alopecia associated with deficiency or excess of thyroid secretion, and the variety known as alopecia areata.

Severe loss of hair may be a sequel of many acute infections, *e.g.* typhoid fever, pneumonia, the exanthemata, and particularly erysipelas, even though the scalp itself be not actually the site of inflammation; it may also follow child-birth, surgical operations, severe injuries, and profound mental shock. But the widespread influenzal infection, that has raged during the past two years, has had a peculiarly malignant effect upon the hair, even compared with previous epidemics of the disease, and we may therefore take post-influenzal alopecia as a type of that resulting from acute infection.

*Post-influenzal Alopecia.*—Apparently even very mild attacks of influenza may cause a subsequent abnormal fall of hair, but the most severe cases of alopecia are seen in patients who have developed pneumonia as a complication, or who have had prolonged fever and bronchitis. Out of a large number of cases seen by the writer early in the year, a very considerable proportion gave a history of influenza in November last, in which month the severity of the disease and the liability to complications were particularly marked.

In the great majority of cases there is a definite latent period between the influenzal attack and the time at which the loss of hair becomes noticeable; the length of this latent period varies, but is usually from four to eight weeks. In a few instances, however, it is much shorter, and in one patient, an Indian medical student, who had a very severe attack with much bronchitis and some broncho-pneumonia, the hair began to fall in handfuls before his temperature had reached the normal.

In order to understand the reason for this latent period, it is necessary to realise what takes place when hair is normally shed. Every hair, of course, grows from, and is at first attached to, a hair-papilla, and during this stage is consequently termed a papillary-hair; if such a hair be forcibly pulled from its papilla, and examined under the microscope, it will be seen to have a bulb-shaped end, which is hollowed out at the point of attachment to the papilla, and the medullary portion can usually be traced from root to tip. In time, however, the hair leaves its papilla, and becomes what Unna called a "bed-hair"; at this stage it is attached to the mid-follicle region, from which it is eventually detached and falls. Bed-hairs may be obtained for examination either by gentle traction from the scalp or from the hair-brush; under the microscope it will be seen that the bed-hair has neither cuticle, root-sheath, nor medulla at its root, which, instead of being bulb-shaped, is merely rounded off. In fact, the difference in appearance of the root of a papillary-hair and that of a bed-hair can easily be recognised with the naked eye.

When an animal moults a large number of hairs leave their papillæ, become bed-hairs, and then rapidly fall; very probably this sudden transformation of a number of papillary-hairs into bed-hairs in animals is dependent on some change in the activity of certain endocrine glands, brought about partly, perhaps, by a seasonal change of temperature, and partly in connection with the breeding season. In human beings the fall of hair that occurs after illness is evidently due to the same phenomenon—namely, the rapid and simultaneous transformation of a large number of hairs from papillary to bed-hairs, and the varying latent period referred to probably depends on the length of time the bed-hairs remain attached to the walls of the follicles before they actually fall. In post-influenzal cases it would seem that the more severe the attack the shorter the latent period.

The question now arises as to the exact cause of the sudden transformation of papillary-hairs to bed-hairs, which is responsible for the "defluvium capillorum" following acute illness, child-birth, surgical operations, etc. Most authors ascribe it to "interference in nutrition," an obviously vague and unsatisfactory explanation. In acute infections the change is probably chiefly due to the direct action of bacterial toxins on the papillary portion of the hair, and there is little doubt that the toxins of some bacteria have a selective action on the hair, particularly, it would seem, those of certain strains of streptococci, for even a brief attack of erysipelas will frequently cause a far greater loss of hair than a prolonged typhoid fever, and in the writer's opinion the probable explanation of the severity of the alopecia met with in the influenzal epidemic is that a secondary streptococcal infection, often of great virulence, so frequently occurred.

Loss of hair, however, following normal labour or an uncomplicated surgical operation, cannot be thus explained. Anæmia may account for a certain number of these cases, for we can understand that an insufficient supply of oxygen to the papillæ would interfere with the proper nutrition of the cells of the papillary portion of the hair, and it is certainly true that in anæmic states the administration of iron, even without local treatment of the scalp, may be followed by an abundant new growth of hair.

But there is another factor, which may play a part in the etiology both of febrile and afebrile cases of alopecia—namely, a disturbance of the normal functions of the endocrine glands; of these the thyroid and the sex-glands seem to have the greatest influence on the growth and nutrition of the hair,

☞ Previous articles appeared on May 17, June 7, July 12 and August 23, page 519.

**The New Dermatology**—(*continued*).

although how this influence is excited is not very clear. The thyroid will alone concern us here.

*Alopecia associated with abnormalities of Thyroid Secretion.*—It is an interesting fact that loss of hair, leading sometimes to complete alopecia, occurs both with excess and deficiency of thyroid secretion. We thus find that it is a symptom of both myxœdema and of Grave's disease.

The following case illustrates the association of alopecia with thyroid deficiency:—

*Case I.*—A girl, aged nineteen, was brought to hospital by her mother for rapid fall of hair. She is a first-born, and only living child. Of the other three children, one died eight days, another eight weeks after birth, and the last was still-born; there were no miscarriages. The patient has always been fat, and mentally childish, and for the past six years has been very deaf. Menstruation did not begin till the nineteenth year, and she had long suffered from obstinate constipation. Her hair was luxuriant until January of this year, when it began to fall rapidly. When seen by the writer she presented obvious signs of thyroid deficiency. The face was broad, and the supra- and infra-orbital "pads" were well shown. The hair, both on scalp and eyebrows, was very scanty, and the tissues of the scalp felt thickened and œdematous when pinched up between the fingers. She was evidently infantile in her mind. The Wassermann reaction was negative. She was given thyroid in small doses, which were gradually increased until the apparently optimum dose was found. New hair soon began to grow, and she now has a thick crop on her scalp, and also on her eyebrows; her mother has also observed great improvement in her general health and in her mental state.

Well-marked cases of myxœdema are usually detected, but the slighter degrees of thyroid deficiency often escape notice. The writer has under his care now two girls in the early twenties, who came complaining of falling hair. They had already been given stimulating lotions for the scalp, but the hair continued to fall. Their appearance is strikingly similar, their faces being unnaturally fat and broad, with thickened pads above the inner halves of the orbits and over the malar bones; their scalps show no sign of pityriasis or seborrhœa, but feel thickened when pinched between the fingers. When first seen their pulse-rates were both below sixty. In both cases small doses of thyroid have resulted in a new growth of hair.

In Grave's disease excessive fall of hair is probably a constant symptom, and a number of cases have been recorded of alopecia universalis associated with this disease. The following case is of great interest in this connection:—

*Case II.*—A girl, aged twenty-three, came under the writer's care early in this year on account of complete baldness, involving the scalp, eyebrows, and eyelids. The hair of the scalp had begun to fall out three years previously, and she became completely bald in the summer of 1918, at which time she also lost her eyebrows and eyelashes. Examination showed the scalp to be smooth and shiny, and, in contrast to the cases of thyroid deficiency, the scalp-tissues felt thin and atrophied when pinched between the fingers. The thyroid gland was much enlarged; there was exophthalmos, and tremor of the fingers; and the pulse-rate was 130-140. The patient presented the flushed appearance so characteristic of Grave's disease, and her hands were continually moist.

Various kinds of stimulation were applied to the scalp without avail, and she was given kathodal labile galvanism for fifteen minutes thrice a week for three months without any success. It was then observed that she had very septic tonsils. These were removed on September 23; in less than a fortnight the thyroid was markedly diminished in size, and at the present time it is normal, the exophthalmos has disappeared, the pulse-rate is below 100, and a fine growth of hair has appeared on her scalp and eyebrows.

Whether the hair will continue to grow remains to be seen, but there can be little doubt that in this case the symptoms of hyperthyroidism were due to chronic toxæmia from infected tonsils, the infecting organism being a streptococcus. The problem is whether the loss of hair was dependent on the excess of thyroid secretion, or whether it was due to the direct action of bacterial toxin.

To return to post-influenzal alopecia, the writer has observed that nearly all the most severe cases show enlargement of the thyroid, tachycardia, tremor, excessive sweating, and the characteristic flushing of hyperthyroidism. Dr. Hector Mackenzie states that a number of cases of Grave's disease appear to have followed an attack of influenza, and fatal cases in the recent influenzal epidemic have almost always shown enlargement and intense congestion of the thyroid gland. Microscopical examination of the thyroid in one such case showed appearances closely resembling those seen in an early case of Grave's disease.

The writer was at first inclined to the view that post-influenzal alopecia might be due, partly at any rate, to the hyperthyroidism that obtains in these cases, but it would seem far more probable that both the alopecia and the hyperthyroidism depend directly on bacterial infection, and, though the actual causal organism of influenza is still a matter of dispute, the micro-organism responsible for these complications is probably a streptococcus.

(*To be continued.*)

